# Glances at the Hospitals

**Published:** 1900-05-05

**Authors:** 


### GLANCES AT THE HOSPITALS.

### BRADFORD ROYAL INFIRMARY.

The annual report for 1899 shows that 15,054 patients were treated during the year. The population of Bradford is about 216,000, so that one-fourteenth of the inhabitants of this town availed themselves of gratuitous medical advice. Something must be deducted for those cases sent in from outside the municipal boundary, but more would have to be added for the numbers treated in the workhouse infirmary, which probably contains as many beds as the Royal Infirmary does. And the Bradford Children's Hospital contains 60

beds; the Eye and Ear Hospital 41 beds. Of the total number of patients 2,017 were in-patients, 6,716 were out-patients, 2,555 were treated in their homes, 3,476 were casualties, and 290 were dental cases. Contrasting these numbers with those of 1898, it is pleasing to be able to record that there has been a considerable diminution in the in-patients, the out-patients, and in the casualties or minor accidents; and in the report for 1900 we shall hope to find that there has been a still further reduction, especially in out-patients. A revised wages list has been fixed as a guide to subscribers issuing recommendations. The daily average number of in-patients was 169, and the average stay in the infirmary was one month. The death-rate among the in-patients was 10·1 per cent., but a considerable proportion died within twenty-four hours of admission. The dispensary medical officers paid 25,576 visits to patients in their own homes. The total sum received on the current account was £8,203. Of this sum £2,646 was derived from subscriptions, £3,208 from the Joint Hospital Fund. The cost per bed occupied was a little over £54, and the total expenditure was £10,702. There was a balance due to the bank of £278. These figures show a deficit of £2,400, and this was met by legacies and donations and by £1,500 from the Mayor's Jubilee Fund. But the larger part of the latter sum had to be used for defraying some of the cost of the nurses' home. As the governors remark, there is still great need for an increase in the permanent annual income of the infirmary. Two conspicuous figures at one time attached to the infirmary have passed away during the year. These were Mr. R. H. Meade and Mr. H. Spencer, both of whom held the post of honorary surgeon. The present medical and surgical staff consists of Drs. Alexander, Major, Goyder, Bell, Meade, Terry, Campbell, Crowley, Denby, Roberts, Horrocks, Althorp, Barber, Wood, Wilmot, Honeybride, Crawford, Enrich, Rabagliati, and Brouner. The resident medical and surgical staff consists of Drs. Chapman, Brown, Earnshaw, Robson, and Lankester.

### THE ROYAL INFIRMARY, ABERDEEN.

The managers of this hospital held their annual meeting on February 14th. Lord Provost Fleming, the president, occupied the chair, and delivered some very clear and sensible introductory remarks, in which he pointed out that there was an improvement in the funds of the institution, and that the managers would soon be able to make both ends meet. In the absence of the chairman of the board (Colonel Allardyce) the annual statement of the affairs of the infirmary was given by Mr. Kyd with much clearness of expression and mastery of detail. He reminded those present that it was the 160th annual meeting of the managers of the Aberdeen Infirmary, and that Hospital Sunday was instituted in their city as early as 1764. The institution is, therefore, no parvenu. During the year a direct personal endeavour to raise the subscriptions was made by a deputation of the board. Many private individuals and others connected with the churches in Aberdeen were waited on with the happy result that the subscriptions and collections were higher during 1899 than for many years, and there is a confident hope that they will be higher still in 1900. Last year the subscriptions reached £1,515, and the donations £1,251; but we do not like to find it recorded that the workpeople's subscriptions fell off slightly during the year, and amounted only to £620. There was, perhaps, never a time when wages were so high, provisions so low, and taxation so slight for that class as at present, and surely an effort should have been made by them to increase their annual contributions. From all sources the hospital received an income of £10,118, and it expended £10,903; but the latter sum includes the interest of borrowed capital, there being upwards of £24,000 of the building debt still unpaid. The capacity of the infirmary is given at 235 beds, the total cost of the building and furniture was £74,000, so that the cost per bed works out at £315. The Edinburgh Infirmary cost £445 per bed. An average of 202 beds were in daily occupation at the Aberdeen Infirmary, and the annual cost per bed occupied was £48, that for Edinburgh and Glasgow being between £8 and £12 more, but Mr. Kyd was careful to point out that efficiency had not been sacrificed to economy. We are glad to have Mr. Kyd's opinion that the charity is not often abused by those who could afford to pay for medical advice, and the admirable plan is adopted of keeping a register of all patients admitted, showing their occupations and giving their addresses. If it be one manager's duty to read and check this list and give orders for stringent inquiries in suspicious cases much good would flow from it. As an additional safeguard, subscribers ought to be notified that tickets should not be given to patients whose wages exceeded a certain amount. Undoubtedly the chief abuses of hospitals rise in the out-patients' departments. There were 2,516 in-patients treated during 1899, and the average duration of residence was 29 days. This number shows that there was a slight decrease of in-patients. On the other hand, there was a regrettable increase of the out-patients from 5,692 to 6,625. The deaths were 179. The population of Aberdeen is about 113,000; and the above figures show that over 9,000 patients received gratuitous medical advice in the Royal Infirmary; and if we add those treated in the other hospitals in the city we get a total of something like 15,500, showing that rather more than one in eight of the population resorted to this form of aid. A number not obtainable by us must be deducted from the above for those patients admitted from the county, and the number treated in the workhouse infirmary, also not at hand, should be added. It is proposed to raise the number of managers from 9 to 11. As a rule, the smaller the board the better and more expeditiously will the work be done; but in the case of the Aberdeen Infirmary there are good reasons given for the election of two additional members. A sensible proposal was made by Mr. Leslie, and agreed to, that the minutes of the meeting should be printed in full and circulated among the members. The medical staff consists of Drs. Smith, Rodger, Fraser, Finlay, Edmond, Gordon, Lister, MacIntosh, Will, Ogston, Garden, Booth, Riddell, Sinclair, Marnock, Grey, Usher, Stephenson, Crombie, and Cromer. Dr. Angus is the medical superintendent, and he has four assistants.

